# Co-designing a pedagogical framework and principles for a hybrid STEM learning environment design

**DOI:** 10.1007/s11423-022-10114-y

**Published:** 2022-05-17

**Authors:** Tiina Mäkelä, Kristóf Fenyvesi, Marja Kankaanranta, Dimitris Pnevmatikos, Panagiota Christodoulou

**Affiliations:** 1 Finnish Institute for Educational Research, University of Jyvaskyla, Jyväskylä, Finland; 2 Department of Primary Education, University of Western Macedonia, Kozani, Greece

**Keywords:** Focus group, Learning environment, Participatory co-design, Pedagogical design principles, Pedagogical framework, STEM

## Abstract

The importance of engaging and effective learning environments for science, technology, engineering and mathematics (STEM) has been internationally recognised. However, no comprehensive pedagogical frameworks exist that support STEM learning environment design. In this study, a pedagogical framework and principles for STEM learning environment design were created based on participatory focus groups involving 10–18-year-old students, teachers, school directors, parents, university students and STEM professionals. Representatives of key stakeholder groups in Belarus, Finland, Germany, Greece and Spain (total *n* = 132) were invited to focus group discussions in which their wishes related to the pedagogical framework were collected. A second focus group discussion session, engaging the same stakeholder groups (total *n* = 137), was implemented to validate the framework. A final review for the framework and its design principles was conducted in online focus group sessions, involving 20 experts in curriculum, STEM, educational policy and/or educational technology from all participant countries. The co-designed framework, which is strengthened by the research literature, entails the following design principle categories: (1) General principles, (2) Cross-curricular skills, (3) Ways of teaching and learning, (4) Socio-emotional aspects and (5) Educational compatibility. The design principles created in this study have been employed in developing a hybrid (virtual, physical, formal, non-formal and informal) STEM environment, but they can be employed in any (STEM) learning environment design. Instead of focusing on singular design principles, we recommend considering a wide range of different design principles in order to support multiple ways of teaching and learning and to develop both subject-related and cross-curricular competencies.

## Introduction

The importance of engaging and effective learning environments for science, technology, engineering and mathematics (STEM) has been internationally recognised (Struyf et al., 2019). There is a need for learning environments that raise interest and motivation towards STEM studies and careers (Loukomies et al., 2013; Salmi et al., 2021) and better connect STEM competencies to cross-curricular, so-called twenty-first century skills, as well as to future workplace skills (Jang, 2016; Struyf et al., 2019). It is also seen as vital to bridge formal school learning with out-of-school, non-formal, e.g. visits to science centres, and informal, e.g. free time activities, learning (Eshach, 2007) in both virtual and physical environments (Bumbacher et al., 2018). Particularly due to difficulties in organising education during the COVID-19 pandemic, more attention has been given to designing hybrid or blended learning environments that combine face-to-face teaching and learning interactions, physical tools and environments with technology-enhanced teaching–learning interactions in virtual environments (Graham & Allen, 2005; Kali et al., 2009), and which connect formal, non-formal and informal learning. However, the creation of frameworks that gather and represent design principles (Kali, 2006; Warr et al., 2020), i.e. organisational units for synthesising design knowledge (Kali et al., 2009), to guide the design of hybrid STEM learning environments is much needed.

Employing different stakeholder groups’ experience and expertise in designing tailor-made learning environments through a participatory design is commonly viewed as beneficial (Kensing & Blomberg, 1998). For instance, design-based research stresses the importance of collaborating with participants and researchers throughout the design process (Wang & Hannafin, 2005). As stated by Kali et al. (2009), it is important to be sensitive to users’ needs and requirements in the design process. Previous studies suggest that integrating the perspectives of students, teachers and designers in participatory learning environment design can improve the design quality (Könings et al., 2014). Particularly, the role of teachers as designers and their knowledge base and articulated principles have been considered crucial in the design (McKenney et al., 2015). Teacher involvement may increase the practicality of the design as well as their ownership and commitment for implementation (Kali et al., 2015). Moreover, involving multiple stakeholder groups in participatory design/co-design facilitates the development of ecologically validated learning environments, as wrong assumptions about stakeholders’ needs can be avoided (Cober et al., 2015). In addition to students and teachers, inviting parents or external experts in the design process may be employed to improve connections between home, school and the wider community (see Mäkelä & Helfenstein, 2016). Further, engaging complex multi-stakeholder partnerships in participatory design ensures stakeholders’ engagement towards achieving sustainable changes aimed at societal transformation (Smith & Iversen, 2018).

We argue, however, that in addition to involving different stakeholders in the participatory learning environment co-design per se, their know-how of engaging and effective learning environments can be used as a basis for developing a framework representing learning environment design principles. In addition to stakeholder involvement in the framework development, it is, nonetheless, vital to ground pedagogical framework and design principles in the existing theories and research literature. Thus, learning environment design can be based on deep educational, practical, empirical and theoretical understanding (Bronfenbrenner & Evans, 2000; McKenney & Reeves, 2013). As in the pragmatic approach in design-based research, theoretical considerations aim to inform and improve practice (Wang & Hannafin, 2005). It is, nevertheless, challenging to develop pedagogical frameworks that entail design principles that integrate both theory and various stakeholders’ views in a balanced manner. Hence, this aspect is often omitted when designing learning environments.

Considering (a) the lack of an existing comprehensive pedagogical framework to support the design of a hybrid learning environment for STEM education, (b) the benefits of the participatory co-design approach and (c) the need to ensure the research perspective in the learning environment design, this paper aims at describing how a pedagogical framework and design principles for a hybrid STEM learning environment were iteratively developed based on key stakeholder groups’ contributions and the research literature. The key stakeholder groups consisted of 10–18-year-old students, teachers, school directors, parents, university students and STEM professionals. Specifically, this paper gives a brief overview of (1) the development process and (2) the final framework and its design principles. This study was part of a broader European research project named STIMEY (Science, Technology, Innovation, Mathematics, Education for the Young) funded by the European Union’s Horizon 2020 research and innovation program (2016–2021), and conducted in Belarus, Finland, Germany, Greece and Spain. The project researched and developed a hybrid STEM learning environment for young people aged 10–18. The developed STEM learning environment consists of components such as a social web platform, e-portfolio, serious games, entrepreneurial tools, a digital radio (virtual learning environment) as well as physical socially assistive robots. It connects various stakeholders in shared efforts to engage and increase both female and male students’ interest and motivation in STEM education, innovations and careers from a young age. In addition to STEM subjects, the STIMEY project focused on cross-curricular skills (also named in the literature as transversal skills or competencies, twenty-first century skills or key competences).

In the first phase of the pedagogical framework development, participating Finnish and Greek stakeholders’ wishes about teaching and learning in general, and STEM subjects in particular, were analysed, and the results were discussed in light of the literature (Mäkelä et al., 2017). In the second phase of our study, the analysis was extended to participants in Belarus, Germany and Spain during two rounds of stakeholder involvement in the form of focus group (1 and 2) discussions in all participant countries. In focus group 1, participants were asked to freely express their wishes. The purpose of focus group 2 was to confirm if their wishes were adequately considered in the initial framework version (see also Pnevmatikos et al., 2021). In focus group 2, some new participants also evaluated the results. In this phase of our study, in addition to stakeholders’ wishes in relation to teaching and learning in general, and STEM subjects in particular, stakeholders’ responses regarding cross-curricular skills were analysed, and a more extensive body of literature supported the analysis (Mäkelä et al., 2020a). This paper presents the third phase of our study, which consists of a systematic summary of the overall framework development process and the final framework version based on the two rounds of general stakeholder involvement (focus groups 1 and 2), the literature and the final revisions suggested by an international group of experts in curriculum, STEM, educational policy and/or educational technology during focus group 3 sessions. The pedagogical framework and design principles described in this article were developed mainly to support the design of a hybrid STEM learning environment and, thus, reflect the objectives defined for this specific STEM learning environment based on the participatory co-design with various stakeholder groups and supported by the research literature. We argue, however, that the design principles are generic enough and can be adapted to serve as guidelines in the design of different STEM learning environments, in particular, but also any learning environments, in general.

## Methods

This study represents educational design research or design-based research, considering education as a design science and intertwining educational design, practice and theory development (e.g., van den Akker, 2007). As customary in design research (Wang & Hannafin, 2005), the study was conducted in close collaboration between researchers and educational stakeholders. The pedagogical framework and its design principles were initially created with various stakeholders, following a participatory design approach (Könings et al., 2014; Mäkelä & Helfenstein, 2016; Pnevmatikos et al., 2021) and the grounded theory approach (Strauss & Corbin, 1998). Focus group techniques (Cortini et al., 2019; Duarte et al., 2015) were deemed adequate to involve various stakeholder groups in the participatory framework co-design in three phases of the study. The purpose of focus group 1 was to involve stakeholders in pedagogical framework development at a very early stage to enable inclusion of design principles in the framework relevant to key stakeholders. Focus group 2 served, in turn, as a type of member check, that is, informant feedback or respondent validation (Koelsch, 2013). Member checks were completed by presenting the design principles created from the input of focus group 1 to the participants involved in focus group 2. This allowed participants to analyse the findings critically and comment on them, and either confirm their accuracy and completeness or propose ideas for improvement. The overall goal of this process was to provide findings that are credible, authentic and reliable from the viewpoints of the key stakeholder groups of this project.

The design principles created from focus group 1’s feedback and confirmed in focus group 2 were then analysed in light of the research literature to strengthen them based on both empirical and theoretical literature. We searched the literature in different electronic databases, e.g. ERIC, Google Scholar, JSTOR and ScienceDirect, using topics that emerged during focus group discussions, e.g. “personalisation”, “active knowledge construction”, “joy of learning”, as keywords. In addition to this, we aimed at identifying design principles that did not emerge in focus groups but were relevant from the perspective of STIMEY project objectives and also supported by the research literature. Our research group agreed, for instance, to add some principles related to “socio-emotional aspects” as well as to “educational compatibility” in the framework. These design principles were added to the framework before conducting focus group 3, which gave the experts participating in these sessions an opportunity to verify their appropriateness. The purpose of focus group 3 was to invite experts of local curriculum, STEM, educational policy or educational technology and who represented all project countries to review the framework and design principles. This expert feedback was considered in the final framework version to increase the ecological validity of the study.

### Participants

Table 1 presents the participants of focus groups 1 and 2. The participants represented the main stakeholder groups relevant to this project, i.e. 10–18-year-old primary, lower and upper secondary school students, university students, school directors, teachers, parents and professionals working in STEM fields (research, non-profit and for-profit organisations). In this way, we ensured that a great variety of perspectives were considered. The volunteering participants had interests and varying know-how and experience in teaching, learning, parenting, STEM sector, design and use of learning environments. While the STEM learning environment developed in the STIMEY project was not targeted at university students, we chose to invite a small number of them to the FGs. We were expecting that their experiences would be valuable, particularly when designing learning environments that raise the attractiveness of STEM studies and careers prior to higher education. The aim was to include the same participants in focus group 2 that were in focus group 1, but also to invite new participants that would evaluate the results without prior participation in the project. Of the focus group 2 participants, 49% had also participated in focus group 1 sessions. Gender balance was assured in the selection of participants in all focus group sessions.

**Table 1:** Stakeholder groups per each country in focus group 1 and 2 (FG1 and FG2) sessions

Stakeholder groups/countries in Focus group (FG) sessions	Finland *n* =		Germany *n* =		Belarus *n* =		Greece *n* =		Spain *n* =		Total *n* =	
	FG1	FG2	FG1	FG2	FG1	FG2	FG1	FG2	FG1	FG2	FG1	FG2
Primary school students	4	7	11	12	2	1	2	2	4	4	**23**	**26**
Lower secondary school students	6	7	2	6	6	7	2	2	5	5	**21**	**27**
Upper secondary school students	2	2	7	10	2	2	2	3	4	4	**17**	**21**
Teachers	3	3	2	2	3	4	6	6	2	2	**16**	**17**
School directors	3	2	3	0	3	3	3	4	2	1	**14**	**10**
Parents	5	3	3	1	4	3	6	6	4	2	**22**	**15**
STEM professionals	2	2	0	4	2	2	3	2	1	1	**8**	**11**
University students	2	1	2	2	5	5	0	0	2	2	**11**	**10**
Total	**27**	**27**	**30**	**37**	**27**	**27**	**24**	**25**	**24**	**21**	**132**	**137**

Bold values indicate totals per stakeholder groups and per countries

Table 2 presents participants in focus group 3. Experts entailed researchers, school directors, teachers, representatives of non-profit and for-profit organisations, and policymakers with expertise in local curriculum, STEM, educational policies and educational technologies. Many participating experts had expertise in various fields, e.g. local curriculum and educational policy, or local curriculum and educational technology.

**Table 2:** Experts and type of expertise per country in focus group 3 sessions

Country	Number of experts	Type of expertise			
		Local curriculum	STEM	Educational policy	Educational technology
Finland	4	3	1	2	3
Spain	3	2	2	0	2
Germany	3	0	1	0	2
Greece	5	3	2	2	2
Belarus	5	1	2	0	1
Total	20	9	8	4	10

### Materials

Materials for focus group sessions were developed in collaboration with the research partners participating in the STIMEY project, first in English and then translated into local languages by the project researchers from each participant country. The research group carefully discussed the contents of the materials in English in order to have a shared understanding among them and to ensure the quality of the translation for each language.

Focus group 1 sessions: In focus group 1, we collected participants’ wishes related to teaching, learning, and assessment (1) in general, and in relation to (2) STEM subjects and (3) cross-curricular skills. These topics were presented to the participants in slides entailing inspirational images about each topic. Participants’ wishes related to each topic were collected using an online form with open-ended questions. “A wish poem” was considered an adequate technique to involve children and adults in writing about their desires in an open, yet structured, manner (see Sanoff, 2002). The participants were asked to state their desires by finishing the following sentences: (1.1) I wish teaching...; (1.2) I wish learning...; (1.3) I wish assessment...; (1.4) I wish motivation...; (2.1) I wish teaching STEM...; (2.2) I wish learning STEM...; (2.2) I wish motivation towards STEM...; (3.1) I wish professional skills...; (3.2) I wish entrepreneurial skills...; (3.3) I wish creativity...; and (3.4) I wish sustainability....

Focus group 2 sessions: A gamified Kahoot survey tool was used for member check or respondent validation. In addition to serving as a motivational affordance (see Keusch & Zhang, 2017), this tool displays the immediate results for the whole group, which can then be discussed together. The Kahoot survey question used for the validation purpose was: “The presented pedagogical design principles foster learning and motivation towards STEM”. Possible answer options were: 4 = strongly agree, 3 = agree, 2 = disagree or 1 = strongly disagree (see also Mäkelä et al., 2020a). Other questions in focus group 2 were related to different components of the STIMEY learning environment, i.e. platform, games, digital radio, robots, and, thus, were left out of this analysis.

Focus group 3 sessions: An online spreadsheet was first created to collect information on each participant’s expertise, e.g. position, organisation and a short description of the type of expertise. We created and shared an online document, including the most recent version of the pedagogical framework developed based on earlier project efforts. Document parts entailed (1) introduction and instructions for participants, (2) description of each framework category, (3) visualisation of the framework and its design principles, (4) description of each pedagogical principle, learning environment design recommendations and guidelines, and some concrete examples of how each principle was considered in this specific STEM learning environment design, (5) conclusions, and (6) list of main references. After document parts 2–5, there was an empty table where experts could provide their opinions, suggest modifications or additions, and express the appropriateness of specific framework components. In conclusion, general opinions of the framework were elicited, e.g. if something was missing, unnecessary, could be modified, or was difficult to understand, or if some parts overlapped. Experts were also encouraged to use the “insert comment” feature to add comments directly to specific document parts.

### Procedures

Participants’ written consent, and in the case of minors, their parents’ consent, were requested in advance. All focus group sessions were recorded.

Focus group 1 sessions: Lasting approximately one hour, face-to-face focus group 1 sessions were organised in all project countries at primary, lower secondary and upper secondary schools during the 2016–2017 school year. Local languages were used in all sessions. The researchers explained that they would formulate design principles for the STEM learning environments based on participants’ wishes. The participants were given time to discuss each topic before writing their wishes down. While open conversation enabled the elicitation of collaborative ideas between stakeholder groups, writing wishes down enabled expressing oneself without the pressure or anxiety of voicing one’s views in front of others (Duarte et al., 2015). It also gave less extroverted participants a better chance to participate. The participants were told that there were no right or wrong responses. They were, however, encouraged to think about and express their wishes as representatives of their stakeholder group, instead of thinking only about their personal preferences (see also Mäkelä et al., 2017).

Focus group 2 sessions: Lasting approximately one hour, sessions were organised during the 2017–2018 school year. In addition to face-to-face sessions, some sessions were organised as video conferences to facilitate the participation of stakeholders from different locations (see Cortini et al., 2019). Local researchers conducted the focus group 2 sessions in local languages in all participant countries. They presented the initial pedagogical framework and design principles created based on the analysis of focus group 1 data, and discussed these with the participants. A Kahoot survey was used to confirm the framework’s relevance from the participants’ perspectives.

Focus group 3 sessions: Experts proficient in the English language were invited to (1) provide information about their expertise, (2) read and leave comments on the shared online documents, and (3) participate in one of the four international one-hour video conference sessions conducted in English by the researchers during May and June 2019. In each video conference session, there were participants from at least three different project countries.

### Data analysis

Focus group 1 sessions: The data analysis was initiated following the grounded theory approach (Strauss & Corbin, 1998). Instead of specific theories on teaching, learning and assessment (1) in general and in relation to (2) STEM subjects and (3) cross-curricular skills, we aimed to identify participants’ wishes on these topics. To code the data, which were collected via an online form, open coding techniques were employed, and data was broken into meaningful conceptual components. Researchers in all project countries analysed the data in their local language. Additionally, the data were also translated into English to create a shared understanding among the researchers participating in the analysis. The researchers shared their initial codes in English based on the data. They compared, discussed and created example responses for each conceptual component identified in the data. At this point, knowledge of existing learning theories and models was used to support the naming of the conceptual components and grouping them into categories.

In the final coding process, the codes were combined into wider thematic groups to create a final list of codes entailing principles 1.1–1.4, 1.6, 2.1–2.4, 3.1–3.11 and 4.2–4.5, as presented in Tables 3, 4, 5 and 6 in the Results section. The names given for each conceptual component were used to name the design principles included in the pedagogical framework. For instance, citations related to students’ active participation were initially coded under “learner’s active agency”, and citations related to knowledge construction were coded under “learning by constructing or creating knowledge”. For the final round of analysis, these initial codes were merged into a wider thematic group named “active knowledge construction”. During the coding process, it was also noticed that wishes expressed in relation to teaching and learning (1) in general and to (2) STEM subjects and (3) cross-curricular skills highly overlapped. For this reason, a unified code list was created for all sections in the final phase of the analysis, instead of keeping a separate code list for each section. The frequency of wishes coded under one code could thus exceed the number of participants, as some participants repeated the same wish in different sections. After creating the final list of codes, the final round of analysis was conducted based on a shared understanding of the conceptual components among the researchers in all participant countries. Reliability of the final coding was assured by the researcher in charge of the coding process, who revised the codes using English translations. Discrepancies between researchers were discussed and resolved, leading to some final revisions. In this analysis, we focused only on the most frequent wishes (*f* > 10).

Focus group 2 sessions: For the purpose of this paper, the percentages of each kind of response (4 = strongly agree, 3 = agree, 2 = disagree or 1 = strongly disagree) in relation to the statement “the presented pedagogical design principles foster learning and motivation towards STEM” were calculated, which served as a member check/respondent validation for the framework and its design principles.

Focus group 3 sessions: In the analysis of focus group 3, we first added transcribed oral comments from experts to tables in a shared online document, including participants’ written comments. After this, we analysed each comment and considered how they could be applied in the final pedagogical framework. We also provided written responses to each comment from participating experts, indicating how their recommendations were considered and why. After completing the framework development, the framework was shared with the experts participating in focus group 3, who also had a chance to see the online document justifying the final modifications based on the focus group 3 discussions.

## Results

Based on the analysis of the data collected in focus group 1 sessions, we formulated the first version of the design principles, which were grouped into four framework categories, i.e. (1) General principles, (2) Cross-curricular skills, (3) Ways of teaching and learning and (4) Socio-emotional aspects, with each including 4–12 design principles. In focus group 2, after presenting the pedagogical framework created based on the feedback from focus group 1, 94.34% of all the participants (n = 137) in focus group 2 either agreed (50%) or strongly agreed (44.34%) with the statement, “the presented pedagogical design principles foster learning and motivation towards STEM”. This assured the validity and usefulness of the pedagogical design principles for STEM learning and motivation from the participant stakeholders’ perspectives. After focus group 1 and 2 sessions, one additional category, (5) Educational compatibility, was added to the framework, based on the need to consider in the learning environment design different educational systems and practices between the STIMEY project countries. Its selection was also strongly supported by the literature, and its importance was verified in focus group 3. As a result of feedback received during the focus group 3 sessions, the whole framework went through some additional changes.

The framework development process is described in the following subheadings. The first column in Tables 3, 4, 5, 6 and 7 presents the pedagogical design principles. The second column presents which principles emerged as a result of the analysis of focus group 1 (*f* = frequency of wishes related to each principle), were confirmed in focus group 2, and verified or modified based on focus group 3. These tables also present design principles (1.5, 4.1, 4.6–4.9 and 5.1–5.8) that did not directly emerge in focus group 1 sessions and, thus, were not confirmed in focus group 2, but were added to the framework based on project objectives and feedback received in focus group 3. The third column gathers examples from the literature to support the inclusion of each principle. In the final column, short examples are provided to exemplify how each design principle can be applied. More examples can be found in a publication created to guide both educators and developers in the learning environment design and use (Mäkelä et al., 2021).

### General principles

This framework category entails six pedagogical design principles that can be applied generally in the learning environment design. As can be seen in Table 3, most of these design principles emerged in focus group 1 sessions and were confirmed in focus group 2 sessions. In relation to *1.1 Personalisation*, the participants in focus group 1 wished that each learner’s competence levels, learning rhythm, preferences, interests and special needs were considered. This principle was confirmed in focus group 2 and verified in focus group 3. Stakeholders participating in focus group 1 also viewed creating connections between learners’ past, present and future knowledge and experiences as important. Based on the feedback received from participant experts in focus group 3, the principle considering these aspects named *Connectedness* (cf. Mäkelä et al., 2020a) was verified but renamed *1.2 Connectedness with learners’ experiences* to avoid confusion, for instance, with connectedness in reference to the Internet. Both design principles 1.1 and 1.2 are strongly supported by the research literature (see Table 3). A principle called *Learning outside the school* emerged based on the wishes expressed in focus group 1 in relation to, for example, field trips and visits to workplaces, and was initially included in category 3. Ways of teaching and learning. However, based on focus group 3, it was noticed that it overlapped with principle *Bridging formal, non-formal and informal learning environments*, initially included in category 5. Educational compatibility. These principles were merged and included in category 1 (1.3 *Bridging formal, non-formal and informal learning environments*) as a principle considered to be generally applicable in the learning environment design (Table 3).

**Table 3:** General principles (*f* = frequency of wishes by FG1 participants, *n* = 132)

1. General principles	Focus groups (FGs)	Supporting literature	Concrete examples
*1.1 Personalisation:* “Considering each learner’s competence level, rhythm, preferences, interests and special needs”	FG1: *f* = 149 FG2: Confirmed FG3: Verified	Baxter et al. (2017), Hargreaves (2004), Lee and Hannafin (2016), Li and Wong (2019) and Reigeluth et al. (2016)	Choose contents and difficulty level based on personal preferences and level of understanding
*1.2 Connectedness with learners’ experiences:* “Creating connections between learners’ past, present and future knowledge and authentic experiences”	FG1: *f* = 175 FG2: Confirmed FG3: Renamed from “Connectedness”	Lee and Hannafin (2016), Merrill (2002), Novak (2002) and Zimmerman & Bell (2014)	Use e-portfolio to store, reflect and display personal experiences
*1.3 Bridging formal, non-formal and informal learning environments:* “Supporting the connections between formal in-school, and non-formal and informal out-of-school learning environments”	FG1: Learning outside the school *f* = 56 FG2: Confirmed FG3: “Learning outside the school” merged with “Bridging formal, non-formal and informal learning environments”	Eshach (2007), Schwier and Seaton (2013) and Thuneberg et al. (2017)	Display events organised virtually or physically outside the school in an online calendar
*1.4 Versatility in both novel and conventional tools and methods:* “Enabling and supporting the versatile use of both novel and conventional tools and methods for learning”	FG1: Versatility *f* = 91, Novelty *f* = 112, Conventionality *f* = 33 FG2: Confirmed FG3: “Versatility”, “Novelty” and “Conventionality” merged	Atjonen et al. (2011) and de Koster et al. (2012)	Combine online simulations with the experiments in a physical laboratory
*1.5 Flexibility and adaptability:* “Ensuring that the learning environment adapts to varied ways of teaching and learning and that there is spatial and temporal flexibility”	FG1: - FG2: - FG3: Added based on the literature and verified	Kariippanon et al. (2019), Mäkelä et al. (2020b) and Nikolova and Collis (1998)	Allow modifying exercises to fit specific curricular requirements and learner groups
*1.6 Support for teaching and learning:* “Providing support and guidance for teachers and learners in learning environment use”	FG1: *f* = 112 FG2: Confirmed FG3: Renamed from “Teaching and learning aid”	Kirschner et al. (2006), Lee and Hannafin (2016), Rubens et al. (2005), Struyf et al. (2019) and Sun (2016)	Include tutorials for teachers and learners to guide the use of the learning environment

The frequency of wishes coded under one code could exceed the number of participants as some participants repeated the same wish in different sections (see Data Analysis)

Based on the suggestions received during focus group 3 sessions, the principles of *Versatility in tools and methods*, *Novelty in tools and methods* and *Conventionality in tools and methods*, which were initially formulated in focus group 1 based on participants’ wishes, were merged into one principle named *1.4 Versatility in both novel and conventional tools and methods* (Table 3, cf. Mäkelä et al., 2020a). Moreover, principle *1.5 Flexibility and adaptability* was included in the framework after focus groups 1 and 2 based on the literature, indicating that flexible and adaptable learning environments support the design and use of versatile tools and methods (e.g. Kariippanon et al., 2019; Nikolova & Collis, 1998), which was an important STIMEY project goal. Its importance was verified in focus group 3 sessions. Finally, *1.6 Support for teaching and learning* was considered a more accurate expression than the former expression, *Teaching and learning aid* (cf. Mäkelä et al., 2020a) for the principle that emerged in focus group 1 sessions, highlighting the importance of “providing support and guidance for teachers and learners in learning environment use” (Table 3).

### Cross-curricular skills

Table 4 presents four cross-curricular skills that were considered important by focus group 1 participants, and whose importance were confirmed in focus group 2 and verified in focus group 3 sessions. In focus group 1, wishes related to *2.1 Professional skills* entailed, for instance, familiarising oneself with skills needed in future STEM professions and connecting learning with professional life and STEM professions. Wishes related to *2.2 Entrepreneurial skills* included, for example, developing entrepreneurial skills through entrepreneurial games, tournaments and simulations. It was also wished that *2.3 Creativity* or “thinking outside the box” and *2.4 Sustainability skills* be promoted in different activities, including everyday life activities. Based on the feedback received in focus group 3, the principle *Creativity* was renamed *2.3 Creativity and innovation* to gather aspects related not only to creativity but also to innovation (Table 4, cf. Mäkelä et al., 2020a).

**Table 4:** Cross-curricular skills (*f* = frequency of wishes by FG1 participants, *n* = 132)

2. Cross-curricular skills	Focus groups (FGs)	Supporting literature	Concrete examples
*2.1 Professional skills:* “Connecting learning with professional life and STEM professionals”	FG1: *f* = 67 FG2: Confirmed FG3: Verified	Andersson and Andersson (2010), Herrington and Oliver (2000) and Jang (2016)	Enable virtual or physical visits from professionals to schools and visits to their workplaces
*2.2 Entrepreneurial skills:* “Fostering entrepreneurial skills and the creation of both profit and non-profit opportunities”	FG1: *f* = 110 FG2: Confirmed FG3: Verified	Edwards-Schachter et al. (2015) and Raposo and Do Paço (2011)	Use business simulations and organise entrepreneurial tournaments
*2.3 Creativity and innovation:* “Including activities that foster creativity and innovation”	FG1: *f* = 98 FG2: Confirmed FG3: Renamed from “Creativity”	Cachia et al. (2010), Henriksen et al. (2018), Pavlysh et al. (2021) and Thuneberg et al. (2018)	Give learners tools and tasks for creating and presenting creative and innovative solutions
*2.4 Sustainability skills:* “Promoting skills related to social, economic, cultural and environmental sustainability”	FG1: *f* = 73 FG2: Confirmed FG3: Verified	Cebrián and Junyent (2015) and Frisk and Larson (2011)	Include activities for reflecting how to enhance sustainability

### Ways of teaching and learning

A total of 11 design principles related to ways of teaching and learning (Table 5) emerged as a result of focus group 1 discussions and were confirmed as important in focus group 2. The experts participating in focus group 3 generally viewed this category as very relevant in the STEM learning environment design. Principle *3.1 Active knowledge construction* was formulated based on participant stakeholders’ wishes on learners’ active agency, active learning and learning by constructing or creating knowledge. Principle *3.2 Participation and involvement* was related to wishes on participatory, interactive and conversational teaching–learning interactions. These principles were directly verified in focus group 3. As a result of focus group 3, we renamed Collaborative methods (e.g., teamwork, group work, cooperation) as *3.3 Collaborative learning* to make the principle more learning-centred (Table 5, cf. Mäkelä et al., 2020a).

**Table 5:** Ways of teaching and learning (*f* = frequency of wishes by FG1 participants, *n* = 132)

3. Ways of teaching and learning	Focus groups (FGs)	Supporting literature	Concrete examples
*3.1 Active knowledge construction:* “Supporting learners’ knowledge construction instead of teacher-centred memorisation”	FG1: *f* = 85 FG2: Confirmed FG3: Verified	Reigeluth et al. (2016), Scardamalia et al. (2012) and Weinberger and Fischer (2006)	Use a mind map to structure the information
*3.2 Participation and involvement:* “Fostering learners’ participation, involvement and self-expression”	FG1: *f* = 60 FG2: Confirmed FG3: Verified	Anderson et al. (2019) and Graham et al. (2018)	Employ social media tools to participate in sharing ideas and being involved in decision-making
*3.3 Collaborative learning:* “Acquiring knowledge and skills as a result of collaboration and knowledge sharing”	FG1: *f* = 40 FG2: Confirmed FG3: Renamed from “Collaborative methods”	Jang (2016), Kali et al. (2009), Laal and Ghodsi (2012) and Lowyck and Pöysä (2001)	Enable face-to-face and online team, group and pair work
*3.4 Learning through experiences:* “Employing every day or real-life experiences and learning by doing”	FG1: *f* = 68 FG2: Confirmed FG3: Verified	Glahn et al. (2019), Scogin et al. (2017) and Struyf et al. (2019)	Create opportunities for hands-on learning
*3.5 Experiments and inquiry:* “Integrating problem- and inquiry-based learning that foster experiments and discoveries”	FG1: *f* = 88 FG2: Confirmed FG3: Verified	Bumbacher et al. (2018), Hakkarainen & Sintonen (2002), Kali (2006) and Viilo et al. (2018)	Model processes of a scientific inquiry and experiment, and discover matters in virtual or physical laboratories
*3.6 Project-based STEM learning:* “Learning science, technology, engineering and mathematics in a cross-curricular project aimed at solving a real-world problem”	FG1: *f* = 20 FG2: Confirmed FG3: Renamed from “Project-based learning”	Capraro et al. (2013), Jang (2016), Kokotsaki et al. (2016) and Tseng et al. (2013)	Create projects that connect different subjects to gain knowledge applicable in real-life situations
*3.7 Self-regulated learning:* “Enabling autonomous work and awareness of one’s own progress”	FG1: *f* = 43 FG2: Confirmed FG3: Verified	Kankaanranta et al. (2007), Mäkelä et al. (2020b), Reigeluth et al. (2016) and Zimmerman (1990)	Employ tools for tracking one’s own progress
*3.8 Reflective learning:* “Encouraging a reflective approach, deep thinking and understanding”	FG1: *f* = 28 FG2: Confirmed FG3: Verified	Farrell (2012), Hébert (2015), Kankaanranta et al. (2007) and Reigeluth et al. (2016)	Use reflective questions to support reflection on one’s work
*3.9 ICT-enhanced learning:* “Utilising ICT tools to foster learning with technology and learning about technology”	FG1: *f* = 70 FG2: Confirmed FG3: Verified	de Koster et al. (2012), Freeman et al. (2017) and Mäkelä et al. (2020b)	Integrate various types of ICT solutions into the learning environment and use challenges with technology to solve problems
*3.10 Games and gamification:* “Using games and gamified elements in learning”	FG1: *f* = 49 FG2: Confirmed FG3: Verified	Gee (2007), Kiili et al. (2012) and Prensky (2003)	Use stories, imagination, challenges, simulations and rewards in learning
*3.11 Multiple representations:* “Presenting information through both digital and non-digital media and using various types of representations”	FG1: *f* = 24 FG2: Confirmed FG3: Verified	Ainsworth (2006) and Wu and Puntambekar (2012)	Support understanding, interpreting and relating different representations used in learning

In wishes collected in focus group 1, *3.4 Learning through experiences* was connected, for instance, to learning based on authentic everyday or real-life examples, experiential learning and learning by doing. *3.5 Experiments and inquiry* was connected to laboratory experiments, scientific inquiry in learning, discovery learning and problem-based learning. These principles were confirmed in focus group 2 and verified in focus group 3, and they are also strongly supported by the literature (see Table 5). The principle *Project-based learning* gathered wishes related to learning through cross-curricular or transversal projects, phenomenon-based learning or linking different subjects. Based on the recommendations of STEM education experts in focus group 3, we added a reference to STEM to this principle to stress the importance of integrating particularly STEM with other subjects using the project-based approach (cf. Mäkelä et al., 2020a). Principle *3.6 Project-based STEM learning* refers to combining STEM with other subjects in the creation of a concrete outcome to an ill-defined real-life problem (see Capraro et al., 2013; Jang, 2016). Principles *3.7 Self-regulated learning*, e.g. independent, autonomous and self-directed learning, and *3.8 Reflective learning*, e.g. reflection and deep thinking, were wished for in focus group 1, confirmed in focus group 2 and verified in focus group 3 (Table 5).

Participant stakeholders’ wishes related to, for example, the use of mobile technology, virtual glasses, electronic measuring systems, platforms, robots and digital assessment tools led to formulating a design principle *3.9 ICT-enhanced learning*. Wishes related to games and playful, game-like elements led to formulating a design principle *3.10 Games and gamification* and wishes related to the inclusion of visuals, multimedia, audio, simulations and animations led to formulating a design principle *3.11 Multiple representations.* These design principles were confirmed in focus group 2 and verified in focus group 3, and were also strongly supported by the research literature (see Table 5).

### Socio-emotional aspects

Socio-emotional aspects consist of nine principles (see Table 6). Based on proposals received during focus group 3 discussions and supported by the literature, we added principle *4.1 Social and emotional skills.* Principle *4.2 Joy of learning* was formulated based on focus group 1’s wishes in relation to, for instance, enjoyment, learner satisfaction and having fun. Principle *4.3 Extrinsic motivation* gathered wishes related to, for example, rewarding feedback, encouragement, rewards and inspiring learning environments, and principle *4.4 Intrinsic motivation* was related to considering personal interests and desires, for example. These principles were confirmed in focus group 2, verified in focus group 3 and strongly supported by the literature (see Table 6). In focus group 1, participants’ wishes related to, for example, equal treatment of all students, no discrimination and fair assessment were labelled *Justice and equity*. Based on the feedback received in focus group 3, this principle was renamed *4.5 Inclusion, justice and equity* in order to draw more attention to the importance of inclusion (Table 6, cf. Mäkelä et al., 2020a).

**Table 6:** Socioemotional aspects (*f* = frequency of wishes by FG1 participants, *n* = 132)

4. Socio-emotional aspects	Focus groups (FGs)	Supporting literature	Concrete examples
*4.1 Social and emotional skills:* “Exercising social and emotional skills”	FG1: - FG2: - FG3: Proposed as a new principle	Denham et al. (2009), Humphrey et al. (2011) and Mäkelä et al. (2020b)	Support learning social and emotional awareness, empathy and relationship skills in both face-to-face and online environments
*4.2 Joy of learning:* “Creating feelings of enjoyment, accomplishment and satisfaction of learning”	FG1: *f* = 142 FG2: Confirmed FG3: Verified	Anggoro et al. (2017), Rantala and Määttä (2012) and Vihma and Aksela (2014)	Employ playful elements and positive feedback
*4.3 Extrinsic motivation:* “Creating attractive and interesting learning environments for all types of learners”	FG1: *f* = 88 FG2: Confirmed FG3: Verified	Deci and Ryan (2002), Loukomies et al. (2013) and Reigeluth et al. (2016)	Enable collecting achievements, earning badges and receiving certificates
*4.4 Intrinsic motivation:* “Fostering learners’ internal curiosity and inner desire to learn”	FG1: *f* = 53 FG2: Confirmed FG3: Verified	Deci and Ryan (2002), Loukomies et al. (2013) and Reigeluth et al. (2016)	Encourage learners to choose personally relevant tasks
*4.5 Inclusion, justice and equity:* “Ensuring inclusion, fair treatment and equal access for all”	FG1: *f* = 27 FG2: Confirmed FG3: Renamed from “Justice and equity”	Polat (2011), Qvortrup and Qvortrup (2018) and Wayne et al. (2004)	Ensure that all individuals are given opportunities to participate and belong to study groups of their interest
*4.6 Sense of belonging:* “Fostering a sense of belonging, including emotional attachment and caring for others”	FG1: - FG2: - FG3: Added based on the literature and verified	Mäkelä and Helfenstein (2016) and St-Amand (2017)	Use social media tools to enable the creation of and belonging to networks based on one’s interest
*4.7 Teacher–student and peer relations:* “Promoting good teacher–student and peer relations in order to support learning and wellbeing”	FG1: - FG2: - FG3: Added based on the literature; “Teacher-student relations” and “Peer relations” merged	Kiuru et al. (2015), Mäkelä and Helfenstein (2016) and Struyf et al. (2019)	Enable fluent communication by using communication channels that both teachers and learners are familiar with
*4.8 Home-school and wider community relations*: “Fostering good relations and shared understanding between the school, families and the wider society”	FG1: - FG2: - FG3: Added based on the literature; “Home-school relations” and “Wider community relations” merged	Lewis and Forman (2002), Mäkelä and Helfenstein (2016), Santiago et al. (2016) and Zimmerman and Bell (2014)	Provide parents and external experts access to the specific areas of learning environments
*4.9 Safety:* “Assuring that the learning environment is physically, virtually, emotionally and socially safe”	FG1: - FG2: - FG3: Proposed as a new principle	Díaz-Vicario and Gairín Sallán (2017) and Mäkelä and Helfenstein (2016)	Have a clear code of conduct and rules and effective interventions when safety is in danger

The frequency of wishes coded under one code could exceed the number of participants as some participants repeated the same wish in different sections (see Data Analysis)

Principles 4.6–4.8 were added to the framework after focus group 1 and focus group 2, based on the STIMEY project objectives emphasising the importance of connecting various stakeholders in shared efforts to engage and increase students’ interest and motivation towards STEM education and careers (Table 6). *4.6 Sense of belonging*, including emotional attachment and caring for others, was added to this category, as supported by the literature highlighting its significant role in both student engagement and academic success (St-Amand, 2017). Also, the importance of *Teacher-student relations*, *Peer relations*, *Home-school relations* and *Wide community relations* was supported by the literature (e.g. Mäkelä & Helfenstein, 2016). Based on the feedback received in focus group 3, these four principles were reformulated to *4.7 Teacher-student and peer relations* and *4.8 Home-school and wider community relations* to simplify the framework structure (Table 6). Finally, in the STIMEY project, safety was initially considered separately from the pedagogical framework, but the feedback received in focus group 3 and supporting literature made us conclude that *4.9 Safety*, entailing physical, virtual, emotional and social safety (see Díaz-Vicario & Gairín Sallán, 2017; Mäkelä & Helfenstein, 2016), should be part of socio-emotional design principles (Table 6).

### Educational compatibility

This category and its eight principles (Table 7) did not emerge in focus group 1 but were considered essential to take into account in international STEM learning environment design. Its selection was strongly supported by the literature (e.g. Lee, 2003; Mäkelä, 2015), and its importance was verified in focus group 3 sessions. 5.1 *Educational needs and challenges* refers to the need to address contemporary local and global educational needs and challenges. Additionally, considering contextual requirements related to *5.2 Educational system*, *5.3 General curricular goals and contents* and *5.4 Subject-specific goals and contents* were deemed vital in the learning environment design.

**Table 7:** Educational compatibility

5. Educational compatibility	Focus groups (FGs)	Supporting literature	Concrete examples
*5.1 Educational needs and challenges:* “Addressing contemporary local and global educational needs and challenges”	FG1: - FG2: - FG3: Verified	Giménez et al. (2017), Jacobson (2015) and Mikk et al. (2016)	Provide learning activities such as serious games to tackle both low performance or engagement levels
*5.2 Educational system:* “Assuring that the learning environment fits with different educational systems”	FG1: - FG2: - FG3: Verified	Giménez et al. (2017) and Jacobson (2015)	Allow creating and modifying learning content that is in line with the local educational system
*5.3 General curricular goals and contents:* “Matching the learning goals and contents with general local curricular requirements”	FG1: - FG2: - FG3: Verified	Voogt and Roblin (2012) and Wyse and Ferrari (2015)	Use keywords, tags, peer review systems, etc. for indicating the appropriateness of activities within a specific curriculum
*5.4 Subject-specific goals and contents:* “Assuring that the learning environment promotes the acquisition of learning the objectives of the specific subject/s”	FG1: - FG2: - FG3: Verified	Gough (2015) and Marginson et al. (2013)	Describe clearly the subject-specific goals, contents and expected outcomes of each learning activity
*5.5 Organisational practices:* “Fitting learning environment to the local institutions’ everyday organisational practices and operations”	FG1: - FG2: - FG3: Renamed from “Organisational practices and operations”	Hanushek and Woessmann (2017) and Law et al. (2005)	Assure that the learning environment can also be used by teachers with fewer development opportunities or with very large classroom sizes
*5.6 Educational practices at group level*: “Considering differences related to educational practices at group level”	FG1: - FG2: - FG3: Verified	Hanushek and Woessmann (2017) and Mikk et al. (2016)	Enable varied ways of using the learning environment based on local practices related to, e.g., teacher and learner roles or instructional strategies
*5.7 Assessment system and practices:* “Considering local assessment system and practices”	FG1: - FG2: - FG3: Verified	Carter (2019) and Darling-Hammond and Wentworth (2010)	Enable versatility in both conventional and novel assessment types
*5.8 Task and activity types:* “Considering how the learning environment matches locally typical task types and activities”	FG1: - FG2: - FG3: Verified	Nugent et al. (2010) and Sullivan et al. (2015)	Allow creating versatile task and activity types

This category was added to the framework after focus group 1 and focus group 2 based on the project objectives and research literature

It is also important to design learning environments that can be adapted to different *5.5 Organisational practices.* The name of this principle, which refers to local institutions’ everyday organisational practices and operations, was shortened from *Organisational practices and operations* based on feedback received in focus group 3. It was also generally acknowledged that there is a need to consider local *5.6 Educational practices at group level*, 5.7 *Assessment system and practices*, and *5.8 Task and activity types* while simultaneously supporting teachers and learners in novel and varied ways of teaching and learning (see *1.4 Versatility in both novel and conventional tools and methods* and *1.6 Support for teaching and learning*).

## Discussion

This paper presented a summary and results of a participatory co-design of a pedagogical framework and principles for hybrid STEM learning environments. The framework was developed based on three focus group sessions, involving various stakeholders and the research literature. The framework offers a set of principles guiding the design of learning environments that consider cognitive and socio-emotional, subject-related and cross-curricular dimensions of STEM learning. Although the participants were not presented with any specific educational theories when involving them in the framework and design principle development, their contributions were very much in line with sociocultural and socio-constructivist paradigms, inspired particularly by the work of Vygotsky (1978), who viewed social environments and the mediating artefacts as essential for learning. A connection could also be found with Dewey’s (1907, 1916) educational philosophy, which views learning as a learner-centred, active, experiential and reflective endeavour. The design principles developed in this study are particularly in harmony with learner-centred approaches. As described by O’Neill and McMahon (2005), learner-centred views reflect constructivist theories emphasising the importance of places on activity, discovery and independent learning, cognitive theories highlighting the activity, and socio-constructivist theories emphasising the importance of peer interactions in learning (see also Struyf et al., 2019).

Design principles on personalisation and connectedness with learners’ experiences (1.1 and 1.2) are in line with the student-centred design principles emphasising the need to provide opportunities to set personal goals and choices, and understanding the purpose and value of learning objectives (Lee & Hannafin, 2016). Further, in line with learner-centred instructional design principles proposed by Reigeluth et al. (2016), principles 3.1–3.3 of this framework stress the importance of learners’ active participation and collaboration in knowledge construction (see also Laal & Ghodsi, 2012; Lowyck & Pöysä, 2001). These design principles are also in harmony with the principles that Merrill (2002) identified as common in various instructional theories, including the importance of solving real-world problems, activating learners’ existing knowledge as a foundation for new knowledge, as well as applying new knowledge and integrating it into the learner’s world. Likewise, Kali et al., (2009, p. 1067) proposed the following design principles for promoting collaborative learning: (a) “engage learners in peer instruction”, (b) “reuse student artefacts as a resource for further learning”, (c) “provide knowledge representation and organisation tools”, and (d) “employ multiple social-activity structures”.

Principles on personalisation, self-regulated and reflective learning (1.1, 3.7, 3.8) are connected to learner-centred design principles, highlighting the importance of personalising the nature and amount of self-regulation based on learners’ self-regulation skills as well as personalising the ways learners reflect on their learning processes and outcomes (Reigeluth et al., 2016). Self-regulation and reflection can be supported, for instance, by means of e-portfolios (Kankaanranta et al., 2007). Further, principles on the joy of learning as well as extrinsic and intrinsic motivation (4.2–4.4) are connected to learner-centred design principles on affective and motivational factors influencing learning, such as positive emotions, curiosity, enjoying learning and enthusiasm (Reigeluth et al., 2016), and can be supported, for instance, by games and game-like elements (principle 3.10, see also Kiili et al., 2012).

Further, versatility in both novel and conventional tools and methods (principle 1.4) enables combining innovative, more learner-centred learning with traditional, more teacher-directed learning (see de Koster et al., 2012). The importance of guidance and support for teaching and learning (principle 1.6) has also been raised by Kirschner et al. (2006), who identified difficulties in minimally guided instruction in constructivist, discovery, problem-based, experiential and inquiry-based teaching. Likewise, Lee and Hannafin (2016) viewed providing explicit directions and support for learners’ different needs as essential in learner-centred learning environments. The importance of the teacher as a coach or facilitator, as well as the provision of structure, resources, hints and guidance in student-centred STEM learning environments, has also been recognised by Struyf et al. (2019). Furthermore, this design principle is related to pedagogical learning principles named “scaffolding progressive inquiry”, “supporting the active role of tutors”, and “providing tools for structuring and coordinating activities” proposed for web-based collaborative learning environments (Rubens et al., 2005).

In the literature, cross-curricular professional and entrepreneurial skills, as well as creativity and innovation (principles 2.1–2.3), are often viewed as interrelated. As described by Edwards-Schachter et al. (2015), fostering technological (inventions), economic (entrepreneurship) and artistic/cultural creativity in learning requires supporting learners’ abilities to generate ideas, experiment and solve problems in novel ways (see also Jang, 2016). Fostering innovation, in turn, implies guiding the implementation of creative ideas to create economic or social value (Edwards-Schachter et al., 2015). In addition to STEM knowledge and skills, (ill-defined) problem-solving and creativity, STEM professionals (employed or self-employed) need, for instance, social communication, system thinking, and time, resource and knowledge management skills (Jang, 2016). Furthermore, promoting skills related to environmental, social, cultural and economic sustainability (principle 2.4, see also Frisk & Larson, 2011) can be seen as a general goal in all educational activities related to professional life, entrepreneurship, creativity and innovation.

Principle 4.1 Social and emotional skills, which was added to the framework based on focus group 3, entails, for example, emotional and social awareness, emotional and behavioural regulation, empathy, and team and relationship skills (see Denham et al., 2009; Humphrey et al., 2011). Further, “inclusion of all regardless of race, ethnicity, disability, gender, sexual orientation, language, socio-economic status, and any other aspect of an individual’s identity that might be perceived as different” (Polat, 2011, p. 51) is seen as a prerequisite for inclusion, justice and equity (principle 4.5). This also means that everyone’s sense of belonging—teacher-student, peer, home-school and wider community relations—as well as safety (principles 4.6–4.9) are fostered and safeguarded.

While all principles presented in this framework can be seen as supportive to STEM learning, previous literature indicates that some of them are very directly connected to success in STEM. For instance, learning through experiences, experiments and inquiry, project-based STEM learning and multiple representations (principles 3.4–3.6 and 3.11) are at the core of STEM learning. Previous studies indicate (Bumbacher et al., 2018) that it is recommended to employ both virtual, e.g. simulations, and physical, e.g. laboratories, manipulative environments to effectively learn different experimentation strategies (see also principle 1.4). Additionally, connecting learning with STEM professions and collaborating on problem-solving (principles 2.1 and 3.3) have been identified as essential for developing STEM workplace skills (Jang, 2016). Further, it is essential to promote motivation (principles 4.3 and 4.4) towards science learning (Loukomies et al., 2013). According to Struyf et al. (2019), students’ motivation, interest and engagement towards STEM learning and future careers may be improved through learner-centred, cross-curricular, cooperative, problem- and inquiry-based STEM education.

Category 5. Educational compatibility was added to the framework to support the international adaptation of the developed STEM learning environment. As Jacobson (2015) points out, education needs to viewed as a complex system consisting of elements such as different stakeholders, organisational levels, contexts, as well as different educational needs and challenges. One should not assume that learning environments designed for one educational system are adequate in another system without any adaptation (Spyrtou et al., 2017). We argue that particularly versatility in both novel and conventional tools and methods as well as flexibility and adaptability (principles 1.4 and 1.5) are needed when designing learning environments that adapt to different educational systems, curricula, practices, assessment systems and so on (principles 5.2–5.8). With regards to educational needs and challenges (principle 5.1), events such as the COVID-19 pandemic show that, in addition to local educational challenges, global challenges in education, for example, in relation to organising hybrid or online learning, need to be tackled. In addition to flexibility and adaptability in time and spaces, ICT-enhanced learning (principle 3.9) enables, for instance, combining onsite and online learning (Mäkelä et al., 2020b).

Both the results of focus group sessions and previous literature suggest that design principles are highly interrelated. For instance, general principles on versatility, flexibility and support (1.4–1.6) can be seen as enablers for considering other design principles, such as addressing both local and global educational needs and challenges (principle 5.1). Connecting formal STEM studies with learners’ experiences in non-formal and informal environments (principles 1.2 and 1.3) can be seen as supportive for developing professional skills as well as home-school and wider community relations (principles 2.1 and 4.8). Instead of focusing on singular design principles, it is, therefore, recommended that learning environment design takes into account a wide range of design principles based on the assessment of stakeholders’ wishes and research literature.

## Concluding words

This paper described the development process, namely the exploitation of participatory focus groups supported by empirical and theoretical literature and their outcomes, the pedagogical framework and principles supporting the design of a hybrid STEM learning environment. Based on the analysis of the first round of focus group discussions, whereby various stakeholder groups’ wishes on teaching, learning, STEM and cross-curricular skills were collected, it was possible to formulate the first framework version, which was then confirmed by the same stakeholder groups in the second round of focus group discussions (see Mäkelä et al., 2020a; Pnevmatikos et al., 2021). This framework version was further elaborated based on the literature and expert feedback received in focus group 3. We argue that the iterative participatory framework development combined with the research literature is a good way to create theoretically, empirically and practically valid frameworks and principles for the learning environment design. The stakeholders’ involvement and the focus on a specific hybrid STEM learning environment design ensured that the design principles were applicable to this specific learning environment design and its target groups. Nevertheless, strengthening and extending the design principles based on the research literature augments their general applicability in the learning environment design. However, while many design principles included in this pedagogical framework were already presented in the previous literature, particularly cross-curricular skills and socio-emotional aspects can be considered a novel contribution to existing learning environment design principles.

Despite the comprehensiveness of the framework presented, we are aware that there are some areas that can be deemed essential in the design but are omitted from the framework version focusing particularly on the STIMEY project objectives. Depending on the design focus and objectives, there may be a need to include, for instance, design principles related to the design of physical environments, issues related to health and wellbeing (see Mäkelä & Helfenstein, 2016), cultural and societal concerns, and aspects considered important in the technological design (see Mäkelä, 2015). In relation to STEM learning environment development, a more comprehensive list for “twenty-first century STEM competencies” related to workplace skills (see Jang, 2016) could be provided.

In the future, in addition to focusing on participant stakeholders’ frequent wishes and expert views also supported by the literature, it would be beneficial to analyse individual and minority views to see how each individual’s unique perspective could be considered in the participatory learning environment co-design. Additionally, while each focus group session entailed possibilities for discussion, the data were mainly gathered in written form. Particularly, this may have limited younger participants’ possibilities to express themselves, whereas oral data such as group interviews could be used to ensure that every voice is being heard. Furthermore, we acknowledge that the number of participants in this study was limited. In future research, the pedagogical framework could be further validated through a wide-scale quantitative study involving various stakeholder groups. Likewise, the effectiveness of the design principles guiding the STEM learning environment design should be empirically tested, and the design principles should be validated in relation to a STEM learning environment effectiveness evaluation.

The pedagogical framework and design principles presented in this article were considered in the design and development of the STEM learning environment. The final pedagogical framework, similar to work by Kali (2006) and Kali et al. (2009), also includes more concrete recommendations and guidelines for considering different design principles in the design, and some examples of how these principles were considered in this specific STEM learning environment design have also been published to serve practitioners in this field (Mäkelä et al., 2021). This framework version also entails an additional category for gender inclusion.

The current study has implications not only for designers and practitioners but also for scholars and policymakers, who could employ the pedagogical framework and its design principles to support the design of hybrid STEM learning environments or learning environments in general. Involving representatives of different stakeholder groups from five countries in three rounds of participatory framework development ensures better acceptance and practical applicability of these design principles in different contexts. The design principles that emerged in this process were also strengthened by empirical and theoretical research literature, thus ensuring both their empirical and theoretical soundness. In the future, we envision creating a more comprehensive collection of research-based design principles of which developers and educators designing learning environments could be scaffolded to choose the most suitable principles based on the specific design focus. This could be a continuation of the “Design Principles Database” created by Kali (2006) and Kali et al. (2009).
